# The value of diffusion kurtosis imaging and intravoxel incoherent motion quantitative parameters in predicting synchronous distant metastasis of rectal cancer

**DOI:** 10.1186/s12885-022-10022-7

**Published:** 2022-08-25

**Authors:** Xue Ding, Danqi Sun, Qiuchen Guo, Yeting Li, Hao Chen, Xiaoxiao Dai, Guohua Fan, Yongyou Wu, Guangqiang Chen, Yonggang Li

**Affiliations:** 1 Department of Radiology, First Affiliated Hospital of Wanan Medical College, NO.2 Zheshanxi Road, Wuhu City, Anhui Province 241000 China; 2 Department of Radiology, First Affiliated Hospital of Soochow University, NO.899 Pinghai Road, Suzhou City, Jiangsu Province 215004 China; 3 Department of Radiology, Second Affiliated Hospital of Soochow University, NO.1055 Sanxiang Road, Suzhou City, Jiangsu Province 215004 China; 4 Department of Pathology, Second Affiliated Hospital of Soochow University, NO.1055 Sanxiang Road, Suzhou City, Jiangsu Province 215004 China; 5 Department of General Surgery, Second Affiliated Hospital of Soochow University, NO.1055 Sanxiang Road, Suzhou City, Jiangsu Province 215004 China

**Keywords:** Rectal cancer, Synchronous distant metastasis, Diffusion kurtosis imaging, Intravoxel incoherent motion, Histogram analysis

## Abstract

**Background:**

The incidence and mortality rate of rectal cancer are still high, the metastasis of rectal cancer are main causes of death. The control of the distant metastasis is one of the main concerns in the treatment of locally advanced rectal cancer, but there are few studies on predicting synchronous distant metastasis (SDM) of rectal cancer.

**Method:**

The data of patients with rectal adenocarcinoma confirmed by endoscopic biopsy or postoperative pathology from September 2015 to May 2020 in hospital A (center 1) and hospital B (center 2) were analyzed retrospectively, including age, sex, carcinoembryonic antigen, carbohydrate antigen 19–9, tumor location, tumor length, image staging and characteristics. The average age of the 169 patients consisting of 105 males and 64 females in study is 61.2 years. All patients underwent rectal routine rectal MRI, DKI and IVIM examinations on a 3.0-T scanner. Two radiologists sketched regions of interest (ROIs) on b = 1000 s/mm^2^ DKI and IVIM images to obtain quantitative parameters with FireVoxel manually. We evaluated the difference of histogram analysis, clinical and image data between SDM group and non-SDM group, and evaluated the efficacy of each index in predicting SDM of rectal cancer.

**Results:**

The 90th percentile of f values in the SDM group is lower than that in the non-SDM group (29.4 ± 8.4% vs. 35 ± 17.8%, *P* = 0.005). CA19-9 in the SDM group is higher than that in the non-SDM group (*P* = 0.003). Low and high rectal cancer are more likely to develop SDM than middle rectal cancer (*P* = 0.05 and *P* = 0.047). The combination of these three indexes has a greater area under the curve (AUC) than any one index (0.801 vs. 0.685 (f (90th percentile)) and 0.627 (CA19-9), *P* = 0.0075 and 0.0058, respectively), and its specificity and sensitivity are 80.0% and 71.6%, respectively. When this combination is incorporated into the predictive nomogram model, the c-index is 0.801 (95% confidence interval (CI): 0.730–0.871).

**Conclusions:**

IVIM quantitative parameters combine with CA19-9 and tumor location can better predict the risk of SDM of rectal cancer.

## Background

The incidence of colorectal cancer ranks third among all cancers across the world, and the mortality rate ranks second, of which rectal cancer accounts for about 1/3 [1]. In China, the incidence of colorectal cancer is lower than that in the United States and Britain, but its mortality rate is higher [2]. Metastasis and recurrence of rectal cancer are main causes of death [1, 2]. After the introduction of total mesorectal excision and neoadjuvant radiotherapy and chemotherapy, the success rate of rectal cancer resection has been higher, and the local recurrence rate has been significantly reduced [3–6]. Distant metastasis of rectal cancer is still one of the difficulties in its treatment [7, 8], according to the different time of occurrence and different location of metastasis, distant metastasis of rectal cancer can be divided into synchronous and metachronous distant metastasis. Synchronous distant metastasis(SDM) refers to the distant metastasis which found during baseline examination, metachronous distant metastasis refers to metastasis which found after baseline examination or after total mesorectal excision [9]. And the most common metastatic sites are the liver and lungs [10–12]. There are different treatment principles and methods in synchronous or metachronous metastases. The treatment of SDM of rectal cancer should consider the situation of the primary cancer to choose the treatment sequence and systemic treatment strategy [13]. Surgical resection is the first choice of treatment, as it can significantly improve the survival rate [14–16]. The research results of the latest treatment model for rectal cancer: total neoadjuvant therapy (TNT), found that this treatment model has greater benefits in patients with high-risk and organ-preserving rectal cancer, but its application in patients with distant metastasis of rectal cancer remains to be studied.

The diagnosis of distant metastasis of rectal cancer mainly depends on laboratory and imaging examination. Some studies have found that laboratory values such as carcinoembryonic antigen (CEA) and carbohydrate antigen 19–9 (CA19-9) can diagnose SDM of rectal cancer [17–19], but their positive predictive value is not high, and their significance is limited. Rectal MRI examination is already a routine examination for patients with rectal cancer, while routine screening for distant metastasis of rectal cancer has consisted mainly of chest and abdominal CT examination, abdominal MRI examination and systemic PET-CT examination, but these have some limitations: owing to the subjective factors of the radiologist, there are some misdiagnoses and missed diagnoses [20, 21]; CT examination has radiation damage to human body; MRI and PET-CT examinations are more expensive; and the specificity of PET-CT examination is low. Other studies about rectal MRI examinations have found that T stage [22], lymph node metastasis [23], circumferential resection margin (CRM) [24] and extramural vascular invasion (EMVI) [25] can also predict SDM, but these results can only be exactly known after operation, and it is impossible to accurately judge whether the patient has distant metastasis before treatment, so its presence or absence can not be used to guide the strategy of preoperative treatment.

MRI functional imaging diffusion kurtosis imaging (DKI) and intravoxel incoherent motion (IVIM) have been rarely used in rectal cancer distant metastasis. Yu's study [26] found that the histogram metrics 10th percentile of Dapp (Dapp-10th) can predict the existence of SDM of rectal cancer, area under the curve (AUC) is 0.856, but the sample size of this study was small, clinical and imaging features were not included in the study. The purpose of our study is to explore whether the quantitative parameters of DKI and IVIM combined with clinical-imaging features can predict the risk of SDM in patients with rectal cancer.

## Materials and methods

### Patients

**Fig. 1:**
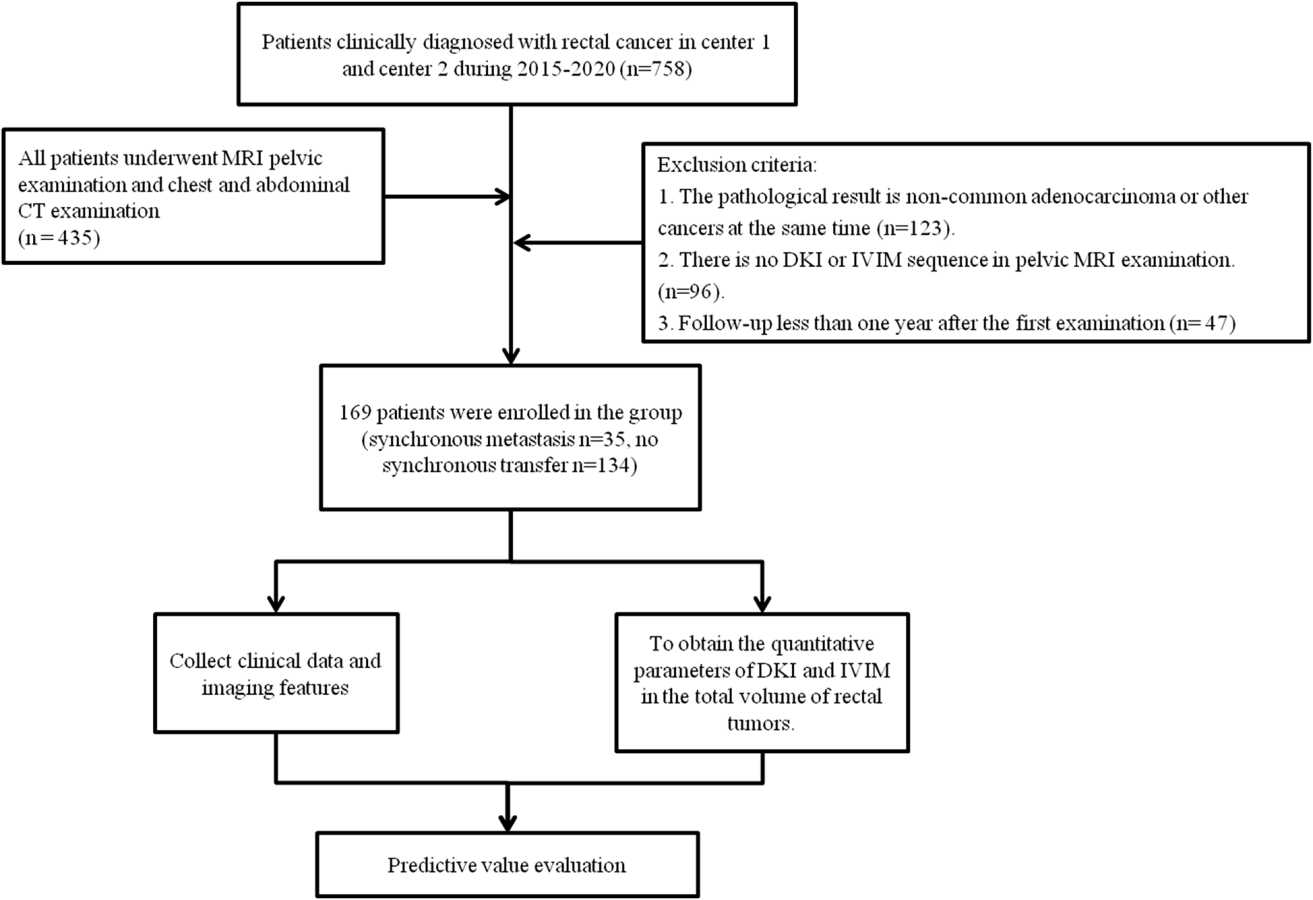
Flowchart of inclusion and exclusion

The data of 169 patients with rectal cancer confirmed by endoscopic biopsy or postoperative pathology from September 2015 to May 2020 in hospital A (center 1) and hospital B (center 2) were analyzed retrospectively. Clinical and imaging data were collected, including age, sex, CEA, CA19-9 and routine MRI, DKI, IVIM images. Inclusion criteria: routine rectal MRI, DKI and IVIM examinations were performed at baseline; neoadjuvant radiotherapy and chemotherapy were not received before baseline examination, exclusion criteria: the image quality of the patient was poor or the tumor was too small to outline the regions of interest (ROIs); the patient had other cancer or the pathological result was not rectal adenocarcinoma; the clinical data were incomplete, the flow chart is shown in Fig. 1.

### Imaging acquisition

All patients underwent rectal MRI with a 3.0-T scanner (Ingenia of Philips Medical Systems, machine 1 and Prisma of Siemens Medical Systems, machine 2) using a 32-channel phased-array body coil in the supine position. Routine rectal MRI, DKI and IVIM examinations were performed in all patients. Scan sequences included sagittal T2-weighted imaging (T2WI), oblique axial high-resolution T2WI (HR-T2WI; the location line was perpendicular to the long axis of the intestinal canal in which the tumor resided), and oblique axial isotropic DKI and IVIM sequences (positioning was the same as for high-resolution T2WI) with respective b values were 0, 1000, 2000s/mm^2^ and 0, 50, 100, 200, 500, 1000 s/mm^2^. The scan parameters are shown in Table 1.

**Table 1:** MRI acquisition parameters

Machine	Sequence	TR (ms)	TE (ms)	Slice thickness (mm)	Slice gap (mm)	Field of view (mm)	Matrix	NSA
1	T2WI	4155	85	3	1	240	300 × 300	2
1	HR-T2WI	4228	85	3	0	260	300 × 289	1
1	DKI(0,1000,2000)	4500	65	3	0	260	160 × 160	2
1	IVIM(0,50,100,200,500,1000)	4500	65	3	0	260	160 × 160	2
2	T2WI	4000	89	3	3	240	300 × 300	2
2	HR-T2WI	4200	101	3	3	260	300 × 300	1
2	DKI(0,1000,2000)	5248	77	5	0	260	104 × 130	2
2	IVIM(0,50,100,200,500,1000)	6000	70	3	0	260	104 × 130	2

*DKI* Diffusion kurtosis imaging, *IVIM* Intravoxel incoherent motion

### Qualitative image evaluation

Two radiologists (with 7 and 5 years of experience in gastrointestinal imaging, respectively) reviewed the first pelvic MR images of all patients together and evaluated the tumor length, tumor location(high = 1, middle = 2, low = 3), T stage in MRI (mrT), N stage in MRI (mrN), CRM in MRI(mrCRM) and EMVI in MRI(mrEMVI). Two other radiologists (with 5 and 4 years of experience, respectively, in chest and abdominal diagnosis) analyzed all the images of each patient to assess the presence of distant metastasis together, and eventually diagnosed by clinical follow-up or pathological results. When the observer could not reach a consensus, another experienced radiologist (with 20 years of experience in thoracoabdominal diagnosis) was consulted for final advice. The evaluation of rectal cancer was based on the 8th edition of AJCC colorectal cancer staging system [27].

### Quantitative image evaluation

**Fig. 2:**
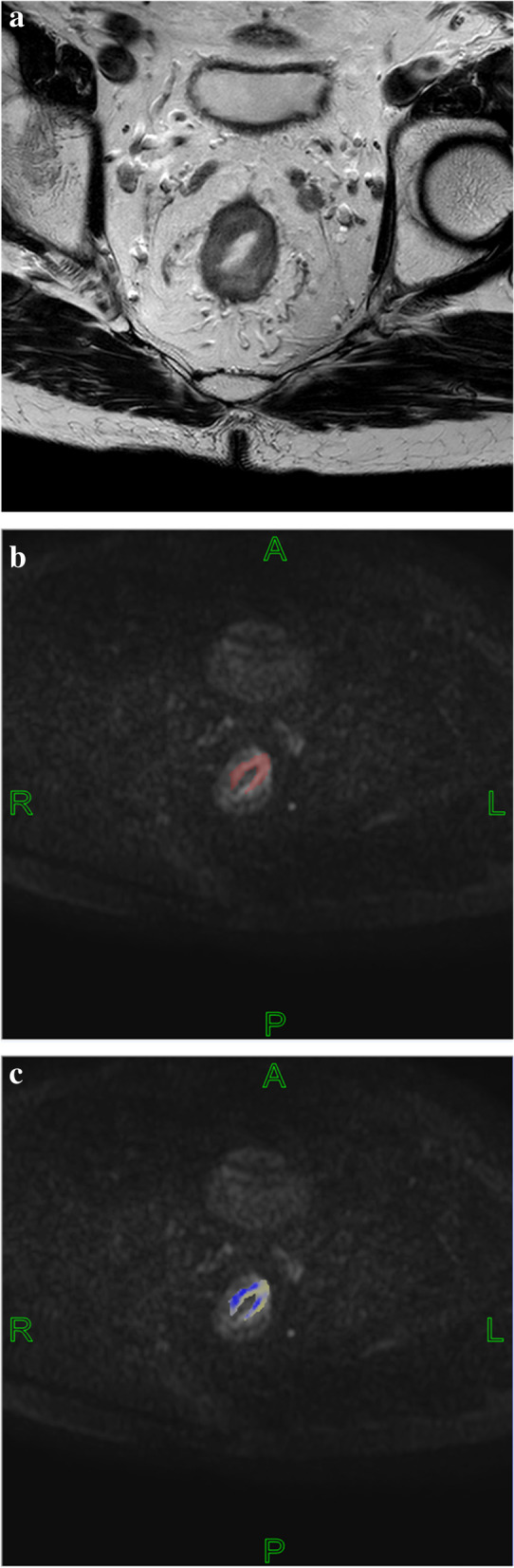
a-c Manual segmentation of rectal cancer in diffusion kurtosis imaging

**Fig. 3:**
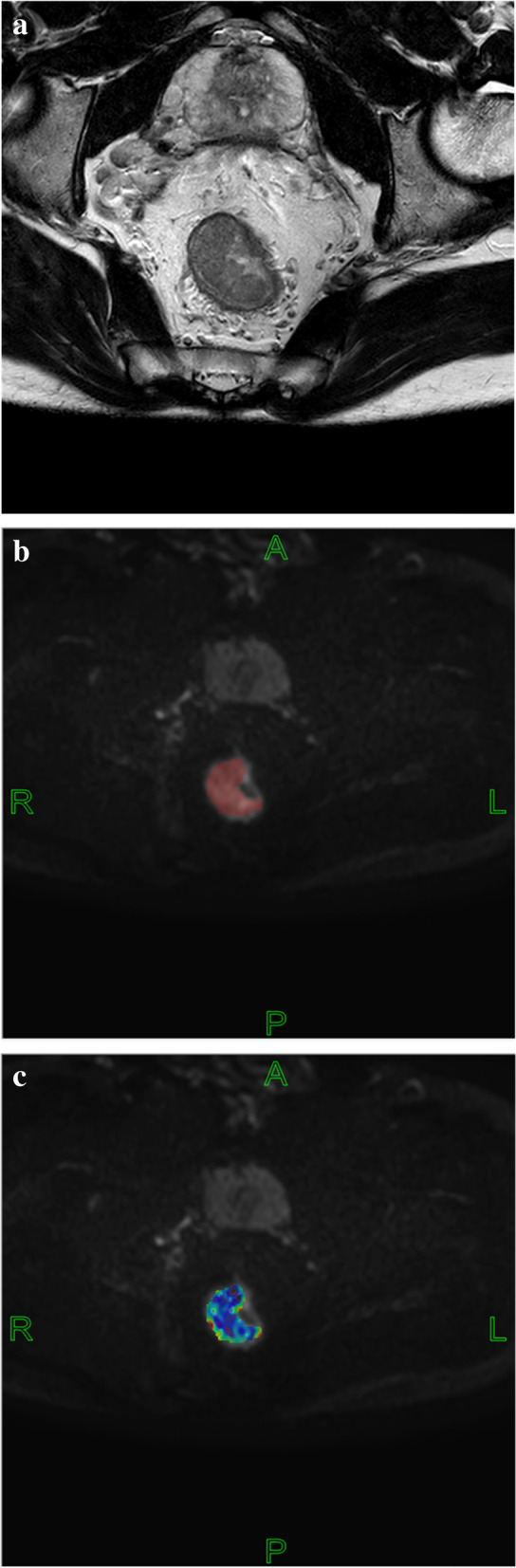
a-c Manual segmentation of rectal cancer in intravoxel incoherent motion

Two radiologists with 2 and 5 years of experience in imaging diagnosis of intestinal tumors used FireVoxel software, combined with HR-T2WI images (Fig. 2a, 3a), to eliminate gas, intestinal contents, cystic areas, etc. [28]. They manually sketched regions of interest (ROIs) with multi layer fusion along the tumor edge on b = 1000 s/mm ^2^ DKI and IVIM images (Fig. 2b, 3b) to obtain a three-dimensional ROI of the tumor. Based on the tumor 3D ROI measurements, we calculated corresponding quantitative parameters according to the diffusion kurtosis model [29] Sb/S0 = exp(-b·D + b^2^·D^2^·K/6) and the intravoxel incoherent motion model [30] S0*(f*exp(-b*Dfast) + (1-f)*(exp(-b*Dslow)), where S0 represents the diffusion-weighted imaging (DWI) signal intensity of a pixel on the MR image when b = 0; Sb represents the DWI signal intensity value of a pixel on the MR image when b = b. D is the non-Gaussian diffusion coefficient, K is the kurtosis coefficient, Dfast is the fast diffusion coefficient, Dslow is the slow diffusion coefficient and f is the perfusion fraction. The corresponding pseudo color map was generated (Fig. 2c, 3c). We evaluated the consistency of the parameters measured by the two physicians. If the consistency was good, then we took the average of the measured values in the ROI outlined by the two as the final data for statistical analysis. MATLAB 2018b and SPSS 23.0 were used for histogram analysis, and we could get average, median, 10th percentile, 25th percentile, 75th percentile, 90th percentile, skewness and kurtosis of the quantitative parameters.

### Statistical analysis

SPSS 23.0, MedCalc 12.1 and R software were used for statistical analysis and graphing. All data are standardized and preprocessed. The normality test and variance homogeneity test were carried out on the measurement data. The data with a normal distribution are represented by x ± s, and the nonnormal distributed data are represented by the median ± quartile. The two-sample t-test was used to compare the count data between groups. After comparing the differences in clinical data and quantitative histogram parameters between the SDM group and the non-SDM group by the Mann Whitney test, we found out main indexes and multivariable comprehensive analysis indexes with bivariate logistic regression analysis. In MedCalc 12.1, receiver operating characteristic (ROC) curves were used to evaluate the efficacy of SDM-related quantitative histogram parameters in identifying SDM of rectal cancer, and the best cutoff value for each parameter was determined based on the ROC curves to calculate the sensitivity and specificity of the value for differential diagnosis. The DeLong method was used to compare the area under the curve (AUC) of different parameters to determine their significance. All the significant variables from binary logistic regression analysis were included in multivariable models and developed predictive nomograms. Harrell's c-index was used to evaluate the discriminant ability of the model. According to the consistency test of quantitative histogram parameters measured by ROI, the intraclass correlation coefficient (ICC) was calculated (0.00–0.20, poor correlation; 0.21–0.40, fair correlation; 0.41–0.60, moderate correlation; 0.61–0.80, good correlation; and 0.81–1.00, excellent correlation). Statistical tests were two sided, and the test level was *α* = 0.05.

## Results

### Patient characteristics

Of the 169 patients, 33 patients received TME after chemotherapy or radiotherapy and chemotherapy, 132 patients did not receive preoperative neoadjuvant therapy, and the other 4 patients only received chemotherapy or radiotherapy and chemotherapy. Thirty-five patients had SDM, including 20 cases of liver metastasis, 10 cases of lung metastasis, 1 case of bone metastasis, 1 case of synchronous liver and lung metastasis, 1 case of distant lymph node metastasis and 2 cases of multiple distant metastasis. Six cases were confirmed by postoperative pathology, and the rest were diagnosed by imaging examination. The detailed features of the patients are listed in Table 2. There is no significant difference in sex, age, mrT, mrN stage or tumor length between the two groups. The levels of CEA and CA19-9 in the SDM group are significantly higher than those in the non-SDM group (*P* = 0.005 and *P* = 0.02). Patients with positive mrCRM or mrEMVI are more likely to have SDM (*P* = 0.006 and *P* = 0.011). High rectal cancer is more likely to have SDM than low rectal cancer (*P* = 0.004).

**Table 2:** Demographic and clinical characteristics of the 167 patients with rectal cancer

Characteristics	non-SDM (*n* = 134)	SDM (*n* = 35)	*P*
Age(year)	63 ± 14	59.49 ± 11.69	0.391
Sex			
Female	53	11	0.378
Male	81	24	
CEA(ng/ml)			
< 10	105	19	**0.005**
≥ 10	29	16	
CA19-9(U/m)			
< 30	107	21	**0.02**
≥ 30	27	14	
Tumor location			**0.012**
1 (high)	42	15	
2(middle)	74	10	
3 (low)	18	10	
1vs.2			0.028
1vs.3			0.371
2vs.3			**0.004**
Tumor length	45.5 ± 22	52 ± 29	0.174
mrT			
T1-2	39	6	0.154
T3-4	95	29	
mrN			
N0	36	8	0.630
N1 + 2	98	27	
mrCRM			
+	104	19	**0.006**
-	30	16	
mrEMVI			
+	105	20	**0.011**
-	29	15	
Center			
1	74	15	0.192
2	60	20	
Machine			
1	70	15	0.323
2	64	20	

*SDM* Synchronous distant metastasis, *CEA* Carcinoembryonic antigen, *CA19-9* Carbohydrate antigen 19–9, *mrT* T Stage on MRI, *mrN* N stage on MRI, *mrCRM* Circumferential resection margin on MRI, *mrEMVI* Extramural vascular invasion on MRI

Tumor location: high = 1, middle = 2, low = 3

### Histogram index analysis of quantitative parameters of DKI and IVIM

The ICCs of D (kurtosis, 25th percentile), K (skewness, kurtosis) and Dslow (average, kurtosis, 25th percentile, 50th percentile) in the histogram indexes of the DKI and IVIM quantitative parameters between the two observers are good correlation, and the ICCs of other indicators are excellent correlation, as shown in Table 3. Although the ICCs of some histogram parameters is not excellent correlation, generally speaking, the consistency between the two observers is good, and the repeatability of this method is good.

**Table 3:** DKI and IVIM quantitative parameter histogram analysis results

Histogram index	D (95% CI)	K (95% CI)	f (95% CI)	Dfast (95% CI)	Dslow (95% CI)
Mean	0.855(0.808–0891)	0.831(0.778–0.872)	0.944(0.925–0.959)	0.964(0.952–0.974)	0.767(0.697–0.823)
Skewness	0.804(0.744–0.852)	0.740(0.663–0.801)	0.906(0.875–0.930)	0.963(0.950–0.972)	0.836(0.784–0.876)
Kurtosis	0.611(0.507–0.698)	0.709(0.625–0.777)	0.835(0.783–0.876)	0.961(0.948–0.971)	0.715(0.633–0.781)
10th	0.849(0.801–0.886)	0.898(0.864–0.923)	0.875(0.834–0.906)	0.999(0.998–0.999)	0.820(0.764–0.864)
25th	0.797(0.734–0.846)	0.869(0.826–0.901)	0.958(0.944–0.968)	0.827(0.773–0.869)	0.794(0.731–0.844)
50th	0.866(0.822–0.899)	0.910(0.881–0.933)	0.948(0.930–0.961)	0.886(0.848–0.914)	0.762(0.690–0.818)
75th	0.868(0.825–0.901)	0.874(0.833–0.905)	0.946(0.927–0.960)	0.921(0.895–0.941)	0.801(0.740–0.849)
90th	0.823(0.767–0.866)	0.906(0.875–0.930)	0.940(0.920–0.955)	0.831(0.778–0.872)	0.816(0.759–0.861)

*CI* Confidence interval

In the histogram index of quantitative parameters of DKI and IVIM, f (kurtosis) in the SDM group is higher than in that the non-SDM group, and the difference is statistically significant (*P* = 0.013). The SDM group have average, 75th percentile, and 90th percentile of f value that are significantly lower than those in the non-SDM group (*P* = 0.012, *P* = 0.004 and *P* = 0.001), as shown in Table 4.

**Table 4:** Comparison of DKI and IVIM quantitative parameter histogram analysis between the synchronous distant metastasis group and the nonsynchronous distant metastasis group

Histogram index	SDM	non-SDM	*Z*	*P*
D mean	1.353 ± 2.187	1.367 ± 0.296	-0.826	0.409
D skewness*	0.675 ± 0.395	0.632 ± 0.532	0.161	0.872
D kurtosis*	0.695 ± 0.937	0.405 ± 1.154	0.727	0.467
D 10th	0.791 ± 0.159	0.788 ± 0.244	0.45	0.653
D 25th	1.010 ± 0.125	0.997 ± 0.235	0.279	0.78
D 50th	1.275 ± 0.297	1.300 ± 0.277	-0.714	0.475
D 75th	1.629 ± 0.26	1.665 ± 0.399	-1.115	0.265
D 90th	2.008 ± 0.302	2.076 ± 0.416	-1.503	0.133
K mean*	0.770 ± 0.096	0.757 ± 0.172	1.554	0.12
K skewness*	-0.010 ± 0.711	-0.015 ± 1.081	-1.009	0.313
K kurtosis*	2.339 ± 3.242	2.202 ± 3.697	-0.116	0.907
K 10th*	0.418 ± 0.464	0.294 ± 0.471	2.081	0.037
K 25th*	0.613 ± 0.177	0.594 ± 0.208	1.806	0.071
K 50th*	0.789 ± 0.092	0.768 ± 0.154	1.507	0.132
K 75th*	0.966 ± 0.116	0.933 ± 0.164	1.466	0.143
K 90th*	1.104 ± 0.187	1.113 ± 0.198	0.345	0.73
f mean	13.08 ± 3.02	14.49 ± 5.19	-2.5	**0.012**
f skewness*	1.086 ± 0.335	1.002 ± 0.414	1.255	0.209
f kurtosis*	1.544 ± 1.316	0.686 ± 1.409	2.477	**0.013**
f 10th	0.21 ± 0.65	0.24 ± 0.97	0.301	0.763
f 25th	2.61 ± 4.66	1.15 ± 4.14	1.558	0.119
f 50th	10.75 ± 3.15	11.30 ± 6.01	-1.203	0.229
f 75th	19.92 ± 5.15	22.11 ± 9.08	-2.892	**0.004**
f 90th	29.44 ± 8.40	35.03 ± 17.79	-3.362	**0.001**
Dfast mean	0.155 ± 0.044	0.163 ± 0.054	-0.683	0.495
Dfast skewness*	1.722 ± 0.555	1.650 ± 0.544	0.372	0.71
Dfast kurtosis*	2.046 ± 2.412	1.616 ± 2.170	-0.175	0.861
Dfast 10th	0.005 ± 0.002	0.005 ± 0.002	-1.288	0.198
Dfast 25th	0.010 ± 0.004	0.011 ± 0.003	-1.288	0.219
Dfast 50th	0.021 ± 0.012	0.025 ± 0.025	-1.438	0.15
Dfast 75th	0.25 ± 0.001	0.25 ± 0.001	-0.081	0.935
Dfast 90th	0.75 ± 0.434	0.75 ± 0.5	0.298	0.765
Dslow mean	0.993 ± 0.179	1.011 ± 0.234	-0.206	0.837
Dslow skewness*	1.020 ± 0.551	1.040 ± 0.663	-0.175	0.861
Dslow kurtosis*	2.448 ± 2.315	2.075 ± 3.140	-0.632	0.527
Dslow 10th	0.637 ± 0.14	0.613 ± 0.170	0.857	0.391
Dslow 25th	0.773 ± 0.125	0.762 ± 0.135	0.291	0.771
Dslow 50th	0.938 ± 0.195	0.947 ± 0.209	-0.194	0.846
Dslow 75th	1.161 ± 0.898	1.187 ± 0.303	-0.361	0.718
Dslow 90th	1.481 ± 0.189	1.486 ± 0.363	-0.811	0.417

*SDM* Synchronous distant metastasis; D: ×${10}^{-3}$ m ${\mathrm{m}}^{2}$/s; Dp:m ${\mathrm{m}}^{2}$/s; f: %;Dt:×${10}^{-3}$ m ${\mathrm{m}}^{2}$/s; *: without unit

**Fig. 4:**
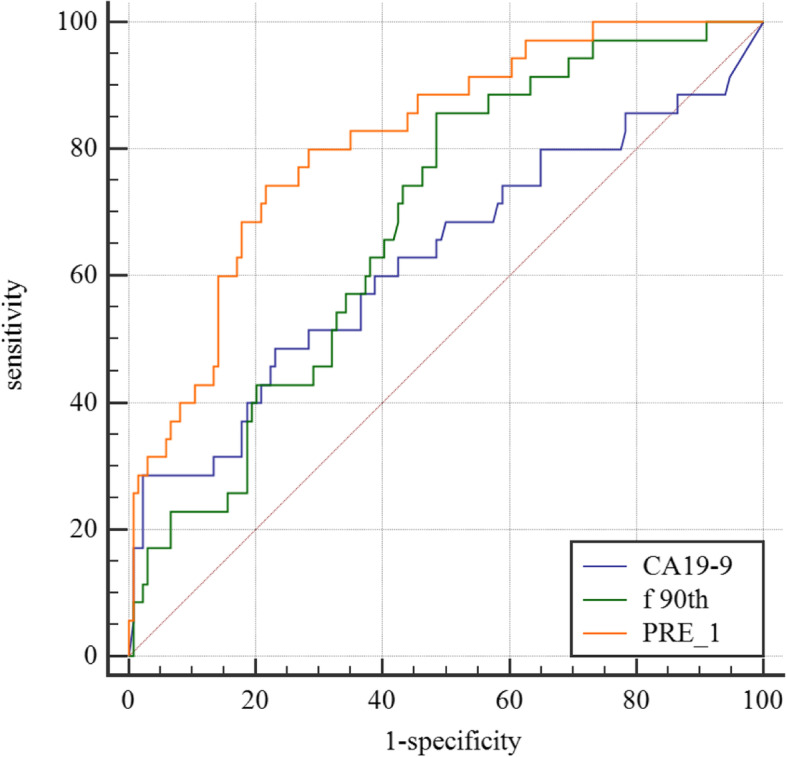
Receiver operating characteristic curves of f (90th percentile), carbohydrate antigen 19-9 (CA19-9) and PRE_1 in the differential diagnosis of synchronous distant metastasis of rectal cancer

**Fig. 5:**
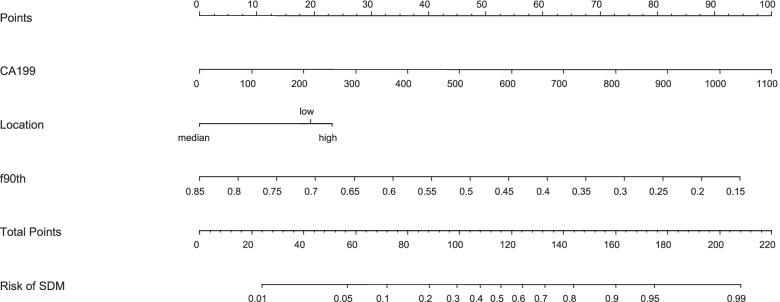
Predictive nomogram for the risk of synchronous distant metastasis (SDM)

The variables with statistically significant differences in Tables 2 and 4 are further screened by multivariable binary logistic stepwise regression, CA19-9, tumor location and f (90th percentile) are significant variables, as shown in Table 5. After calculating the combination of the three variables, the AUC of the multivariable joint analysis index PRE_1 is 0.801, and the sensitivity and specificity are 80.0% and 71.6%, respectively. The pairwise comparison by the Delong method show that the diagnostic efficiency of PRE_1 is higher than that of f (90th percentile) and CA19-9 (*P* = 0.0058 and *P* = 0.0075, *Z* = 2.757 and *Z* = 2.675), as shown in Table 6 and Fig. 4. CA19-9, tumor location and f (90th percentile) are included in the nomogram prediction model, and the C index is 0.801 (95% confidence interval (CI): 0.730–0.871), as shown in Fig. 5.

**Table 5:** Multivariate binary logistic stepwise regression screening results

Characteristics	β	*P*
CA19-9	0.005	0.003
Tumor location		0.072
1 vs.2	-0.965	0.050
3 vs.2	-1.159	0.047
f 90th percentile	-6.739	0.005

*CA19-9* Carbohydrate antigen 19–9, Tumor location: high = 1, middle = 2, low = 3; f: %

**Table 6:** Efficacy of the histogram analysis of DKI and IVIM quantitative parameters in the differential diagnosis of SDM of rectal cancer

Characteristics	AUC	95% CI	Cutoff	Sensitivity	Specificity	Positive predictive values	Negative predictive values	Youden index
CA19-9	0.627	0.550—0.700	184.3	28.57%	97.76%	78.6%	78.4%	0.2633
f(90th percentile)	0.685	0.609—0.754	34.85	85.71%	51.49%	31.6%	93.2%	0.3721
PRE_1*	0.801	0.733—0.858	0.193	80.00%	71.64%	42.4%	93.2%	0.5164

*AUC* Area under the curve, *CI* Confidence interval; f: %; *CA19-9* Carbohydrate antigen 19–9 (U/ml)

## Discussion

Identifying rectal cancer patients with a high risk of SDM before surgery is the key to obtaining an individualized treatment strategy, improving the diagnostic accuracy for patients with suspected metastases and surgically removing the lesions early. In addition, for patients with a high risk of SDM that cannot be detected by imaging, earlier identification would let us take more active treatment measures and give the patient a shorter follow-up period. Gaitanidis [31] developed a nomogram to predict the presence of liver, lung and bone metastases in rectal cancer patients at the same time, he found that rectal cancer patients with synchronous liver metastasis are more likely to have synchronous lung and bone metastasis. It also means the importance of improving the diagnostic accuracy of synchronous distant metastasis of rectal cancer.

We find that there are significant differences in CEA, CA19-9, tumor location, mrCRM and mrEMVI between the SDM group and the non-SDM group. In the IVIM quantitative parameters,there are also difference in f (average, kurtosis, 75th percentile, 90th percentile) between the two groups. Further multivariable binary logistic stepwise regression screening show that only CA19-9, tumor location and f (90th percentile) are significant. The identification efficiency of the three combined indexes PRE_1 (AUC = 0.801) is significantly better than that of f (90th percentile) and CA19-9. PRE_1 model improves the positive predictive value on the premise of ensuring a high negative predictive value. The C index of the nomogram prediction model is 0.801, so its predictive value is good.

Some scholars found that serological indexes such as CEA and CA19-9 can predict SDM in patients with rectal cancer [17–19], this conclusion is also reached in our single factor analysis. However, after binary logistic stepwise regression screening, only CA19-9 has statistical significance in our study, which may have been due to the low positive predictive value of CEA and CA19-9. We find that patients with low and high rectal cancer are more likely to develop SDM. Some scholars found that tumors at the rectosigmoid are more likely to have SDM [31], and other scholars found that low rectal cancer was more likely to recur [32], which may be due to the abundance of blood and lymphatic reflux in high and low rectal cancer. Many scholars found that pathology TN stage, EMVI and CRF status of rectal cancer are also independent predictors of the metastasis and the prognosis of rectal cancer [23–25, 33]. However, we did not include these factors in this study. One reason is that these factors can be diagnosed more accurately in postoperative pathology, and the other is that some patients had received preoperative neoadjuvant therapy, so their results could not be compared with the results of postoperative pathology. There are no significant difference in mrT and mrN stage between the two groups. In single factor analysis, mrCRM and mrEMVI are of great significance in the diagnosis of SDM of rectal cancer, but after further screened by multivariable binary logistic stepwise regression, there are no significant difference. This result is different from some research [22–25]. The reason may be that the diagnostic efficiency of MRI is lower than the pathological gold standard [34].

We find that among the IVIM quantitative parameters, f (90th percentile) has the best diagnostic efficacy. The lower the f values, the lower the perfusion effect. The average and percentile of f value of SDM are mostly lower than that of non-SDM group. We analyze the internal data of the tumor and find that blood supply shortage, which may means that the tumor has external blood supply, such as the blood supply outside the intestinal wall. The tumor with external blood supply maybe have higher possibility to transfer to distant organs. The difference of f (90th percentile) is the largest, possible causes may be that the highest proportion of blood supply still can not reach a critical value, which means that the tumor is more likely to receive blood supply from extra-tumoral vessels. At the same time, the values of Dfast and Dslow in the SDM group are lower than those in the non-SDM group, indicating that the perfusion effect and water molecular diffusion effect are both decreased in the SDM group, but the degree of decrease in perfusion effect is more obvious, the possible reason is that some tumor cells have already spread to distant organs. Some studies also found that the f value is highly useful in differentiating liver metastases of rectal cancer from normal liver tissue [35], pancreatic cancer and normal pancreatic tissue [36]. Some scholars found that D (10th percentile), a quantitative parameter of DKI, can predict distant metastasis, but our study don’t find this. This may be due to the different b values we selected. In clinical work, to reduce the examination time of patients, we set less b values of DKI and IVIM. The combination of f (90th percentile), CA19-9 and tumor location further improved the identification efficiency. The AUC increase from 0.685 (for f (90th percentile)) to 0.801, a statistically significant improvement. Other scholars established a clinical-radiomics combined model based on T2WI images, which could effectively predict the risk of SDM [37], but only histological images were studied and without functional imaging. Hu [38] also found that radiomics models based on T2WI and DWI could be effectively used in assessing liver metastasis in rectal cancer, but the performance of DWI alone models was poor in other models. The possible reason is that DWI has low tissue resolution, which means that it doesn’t have enough features to be extracted. Zhao [39] established a model based on histogram parameters derived from intravoxel incoherent motion diffusion-weighted imaging for predicting the nodal staging of rectal cancer. However,we don’t bring some histogram parameters of Dfast, Dslow, and f value, such as range, energy, total energy, into our research. In the next step, we can incorporate these indicators into research, and perhaps get a better model.

There are also some limitations to our study. One is that only some patients with SDM had a pathological basis for diagnosis, so there may have been missed diagnoses. The other is that there was a certain bias in the included patients, and some patients with incomplete data were excluded.

## Conclusion

In summary, we found that the f value among IVIM quantitative parameters can well predict the risk of SDM of rectal cancer, in addition the diagnostic efficiency is even better when it is combined with CA19-9 and tumor location.

## Data Availability

The datasets used and/or analysed during the current study are available from the corresponding author on reasonable request.

## Abbreviations

AUC: Area under curve
CEA: Carcinoembryonic antigen
CA19-9: Carbohydrate antigen 19–9
CRM: Circumferential resection margin
CI: Confidence interval
CT: Computed tomography
D: Diffusion constant
DWI: Diffusion weighted imaging
DKI: Diffusion kurtosis imaging
Dfast: Pseudo-diffusion
Dslow: Tissue diffusion
EMVI: Extramural vascular invasion
F: Perfusion fraction
IVIM: Intravoxel incoherent motion
ICC: Intraclass correlation coefficient
K: Kurtosis constant
MRI: Magnetic resonance imaging
MDT: Multidisciplinary team
NSDM: No-synchronous distant metastasis
PET-CT: Positron emission tomography
ROC: Receiver operating characteristic
ROI: Region of interest
SDM: Synchronous distant metastasis
TNT: Total neoadjuvant therapy

## References

[1] Bray F, Ferlay J, Soerjomataram I, Siegel RL, Torre LA, Jemal A (2018). Global cancer statistics 2018: GLOBOCAN estimates of incidence and mortality worldwide for 36 cancers in 185 countries. CA Cancer J Clin.

[2] Feng RM, Zong YN, Cao SM, Xu RH (2019). Current cancer situation in China: good or bad news from the 2018 Global cancer statistics. Cancer Commun (Lond).

[3] Engelen SM, Maas M, Lahaye MJ (2013). Modern multidisciplinary treatment of rectal cancer based on staging with magnetic resonance imaging leads to excellent local control, but distant control remains a challenge. Eur J Cancer.

[4] van Gijn W, Marijnen CA, Nagtegaal ID (2011). Preoperative radiotherapy combined with total mesorectal excision for resectable rectal cancer: 12-year follow-up of the multicentre, randomised controlled TME trial. Lancet Oncol.

[5] Sargent D, Sobrero A, Grothey A (2009). Evidence for cure by adjuvant therapy in colon cancer: observations based on individual patient data from 20,898 patients on 18 randomized trials. J Clin Oncol.

[6] Piso P, Dahlke MH, Mirena P (2004). Total mesorectal excision for middle and lower rectal cancer: a single institution experience with 337 consecutive patients. J Surg Oncol.

[7] Sauer R, Becker H, Hohenberger W (2004). Preoperative versus postoperative chemoradiotherapy for rectal cancer. N Engl J Med.

[8] Goodwin RA, Asmis TR (2009). Overview of systemic therapy for colorectal cancer. Clin Colon Rectal Surg.

[9] Adam R, de Gramont A, Figueras J (2015). Managing synchronous liver metastases from colorectal cancer: a multidisciplinary international consensus. Cancer Treat Rev.

[10] Fossum CC, Alabbad JY, Romak LB (2017). The role of neoadjuvant radiotherapy for locally-advanced rectal cancer with resectable synchronous metastasis. J Gastrointest Oncol.

[11] Holch JW, Demmer M, Lamersdorf C (2017). Pattern and dynamics of distant metastases in metastatic colorectal cancer. Visc Med.

[12] Butte JM, Gonen M, Ding P (2012). Patterns of failure in patients with early onset (synchronous) resectable liver metastases from rectal cancer. Cancer.

[13] Van Cutsem E, Cervantes A, Adam R (2016). ESMO consensus guidelines for the management of patients with metastatic colorectal cancer. Ann Oncol.

[14] Kanas GP, Taylor A, Primrose JN (2012). Survival after liver resection in metastatic colorectal cancer: review and meta-analysis of prognostic factors. Clin Epidemiol.

[15] Hur H, Ko YT, Min BS (2009). Comparative study of resection and radiofrequency ablation in the treatment of solitary colorectal liver metastases. Am J Surg.

[16] Kato T, Yasui K, Hirai T (2003). Therapeutic results for hepatic metastasis of colorectal cancer with special reference to effectiveness of hepatectomy: analysis of prognostic factors for 763 cases recorded at 18 institutions. Dis Colon Rectum.

[17] Zhu Z, Zhenghong, Guoweijian (2017). Retrospective study of predictors of bone metastasis in colorectal cancer patients. J Bone Oncol.

[18] Dong H, Tang J, Li LH (2013). Serum carbohydrate antigen 19–9 as an indicator of liver metastasis in colorectal carcinoma cases. Asian Pac J Cancer Prev.

[19] Lin PC, Lin JK, Lin CC (2012). Carbohydrate antigen 19–9 is a valuable prognostic factor in colorectal cancer patients with normal levels of carcinoembryonic antigen and may help predict lung metastasis. Int J Colorectal Dis.

[20] Nordholm-Carstensen A, Jorgensen LN, Wille-Jørgensen PA, Hansen H, Harling H (2015). Indeterminate pulmonary nodules in colorectal-cancer: do radiologists agree. Ann Surg Oncol.

[21] Restivo A, Zorcolo L, Piga S, Cocco IM, Casula G (2012). Routine preoperative chest computed tomography does not influence therapeutic strategy in patients with colorectal cancer. Colorectal Dis.

[22] Yoo HY, Shin R, Ha HK (2012). Does t3 subdivision correlate with nodal or distant metastasis in colorectal cancer. J Korean Soc Coloproctol.

[23] Wu ZY, Wan J, Li JH (2007). Prognostic value of lateral lymph node metastasis for advanced low rectal cancer. World J Gastroenterol.

[24] Liu Q, Luo D, Cai S, Li Q, Li X (2018). Circumferential resection margin as a prognostic factor after rectal cancer surgery: A large population-based retrospective study. Cancer Med.

[25] Sohn B, Lim JS, Kim H (2015). MRI-detected extramural vascular invasion is an independent prognostic factor for synchronous metastasis in patients with rectal cancer. Eur Radiol.

[26] Yu J, Huang DY, Li Y, Dai X, Shi HB (2016). Correlation of standard diffusion-weighted imaging and diffusion kurtosis imaging with distant metastases of rectal carcinoma. J Magn Reson Imaging.

[27] Weiser MR (2018). AJCC 8th edition: Colorectal cancer. Ann Surg Oncol.

[28] Lambregts DM, Beets GL, Maas M (2011). Tumour ADC measurements in rectal cancer: effect of ROI methods on ADC values and interobserver variability. Eur Radiol.

[29] Lu H, Jensen JH, Ramani A, Helpern JA (2006). Three-dimensional characterization of non-gaussian water diffusion in humans using diffusion kurtosis imaging. NMR Biomed.

[30] Le Bihan D, Breton E, Lallemand D, Aubin ML, Vignaud J, Laval-Jeantet M (1988). Separation of diffusion and perfusion in intravoxel incoherent motion MR imaging. Radiol.

[31] Gaitanidis A, Alevizakos M, Tsaroucha A, Tsalikidis C, Pitiakoudis M (2018). Predictive Nomograms for Synchronous Distant Metastasis in Rectal Cancer. J Gastrointest Surg.

[32] Sugihara K, Kobayashi H, Kato T (2006). Indication and benefit of pelvic sidewall dissection for rectal cancer. Dis Colon Rectum.

[33] Park JS, Huh JW, Park YA (2014). A circumferential resection margin of 1 mm is a negative prognostic factor in rectal cancer patients with and without neoadjuvant chemoradiotherapy. Dis Colon Rectum.

[34] Al-Sukhni E, Milot L, Fruitman M (2012). Diagnostic accuracy of MRI for assessment of T category, lymph node metastases, and circumferential resection margin involvement in patients with rectal cancer: a systematic review and meta-analysis. Ann Surg Oncol.

[35] Koh DM, Scurr E, Collins DJ (2006). Colorectal hepatic metastases: quantitative measurements using single-shot echo-planar diffusion-weighted MR imaging. Eur Radiol.

[36] Lemke A, Laun FB, Klauss M (2009). Differentiation of pancreas carcinoma from healthy pancreatic tissue using multiple b-values: comparison of apparent diffusion coefficient and intravoxel incoherent motion derived parameters. Invest Radiol.

[37] Liu H, Zhang C, Wang L (2019). MRI radiomics analysis for predicting preoperative synchronous distant metastasis in patients with rectal cancer. Eur Radiol.

[38] Hu SX, Yang K, Wang XR (2021). Application of MRI-based radiomics models in the assessment of hepatic metastasis of rectal cancer. Sichuan Da Xue Xue Bao Yi Xue Ban.

[39] Zhao L, Liang M, Yang Y, Zhao X, Zhang H (2021). Histogram models based on intravoxel incoherent motion diffusion-weighted imaging to predict nodal staging of rectal cancer. Eur J Radiol.

